# Modification of Konjac Biodegradable Material Using Deacetylation and Reinforcement Process for Its Applications in Food Packaging

**DOI:** 10.1155/2023/5559783

**Published:** 2023-09-19

**Authors:** Supaphada Sonthithaveelap, Rarisara Impaprasert, Worapot Suntornsuk, George Srzednicki

**Affiliations:** ^1^Department of Microbiology, Faculty of Science, King Mongkut's University of Technology Thonburi, 126 Pracha Uthit Rd., Bang Mod, Thung Khru, Bangkok 10140, Thailand; ^2^Food Science & Technology, School of Chemical Engineering, The University of New South Wales, Sydney NSW 2052, Australia

## Abstract

Common konjac flour, especially of low grade, is a waste material produced in large quantities during purification of konjac glucomannan (KGM). It contains impurities, particularly oxalate salts, which irritate and may cause kidney stones. Konjac flour has glucomannan as a main component. Glucomannan is characterized by low crystallinity, high thermostability, and the ability to form a strong gel. Subsequently, glucomannan has good potential for the production of biodegradable material. However, its high-water affinity limits its use in packaging. The deacetylated by thermal forming process and reinforced konjac flour with 15% and 20% of microcrystalline cellulose showed improved water absorption and thermal properties of the specimen. Moreover, the thermal forming process resulted in the reduction of soluble oxalate. Therefore, due to the conditions used in this experiment, the material will be stronger, more waterproof properties, and more highly resistant to temperatures, so it is suitable to be used as a packaging that is environmentally friendly.

## 1. Introduction

Konjac (*Amorphophallus konjac*) is a tuber plant originated from Southeast Asia. It is found in the tropical regions of East Asia from Japan to the South of China and Indonesia [1]. *A. konjac* has been grown in China and Japan for several centuries, and its cultivation is expanding. Currently, *A. konjac* is being investigated as a potential new crop in Western Europe and New Zealand to be utilised in the food industry [2]. In Thailand, 46 species of *Amorphophallus* have been identified. They grow predominantly in the northern and western regions. The main species used for food processing is *Amorphophallus muelleri* (“Buk Nue Sai” or “Buk Khai”), which contains high amounts of glucomannan [3].

Konjac glucomannan (KGM) is a heteropolysaccharide consisting of *β*-1,4 linked D-mannose (M) and D-glucose (G) at a ratio of 1.6 : 1 with O-acetyl group, which affects water absorption and swelling properties [4]. KGM is used as an agent for gelling, emulsifying, stabilizing, and thickening in food products such as sauce, jelly, noodles, and food supplements [5]. These products require purified KGM, making the purification process necessary. The impurified raw product from the tuber of the perennial *Amorphophallus* species is defined as konjac flour (KF) [6]. KF contains mostly KGM, including very small molecules, which are not used in the food industry, and also impurities, including starch, protein, fat ash, and oxalate salts. The oxalate salts can cause kidney stones or skin irritation [7]. These impurities cannot be used in the food industry and are eliminated in the purification process.

In recent years, agricultural materials such as cassava starch, corn starch, or plant pulp have been used to produce biodegradable packaging instead of plastic packaging due to lower cost, availability, biodegradability, and being environmentally friendly. Nevertheless, these biopolymers have properties that are not suitable for packaging such as high-water sensitivity and low thermal stability [8]. Therefore, they are commonly combined with another polymer or a reinforcing agent to improve their properties. The main reinforcing agent is microcrystalline cellulose (MCC), which is nontoxic, biodegradable, and highly stable with suitable mechanical and thermal properties [9]. Besides, MCC has a molecular structure similar to starch and glucose polymer, resulting in a composite with compatibility and strength. For example, soy-based bio-polyurethane with MCC has a high impact strength and thermal stability because MCC acts like a chain extender to produce the strong interaction between composites [10]. The reinforcement with MCC improves the water resistance of thermoplastic starch (TPS) from wheat flour and TPS from cassava starch with polyester. As a result of MCC crystallinity, the water absorption and swelling of starch granules are restricted [11, 12].

KGM is a natural polymer, which is cheap, available, and biodegradable. Furthermore, it has the ability of gel and film forming [13]. There are several studies on biodegradable packaging from KGM and KF such as KGM film with high thermal resistance [14] and edible film from KF and KF with hydrocolloids, which have superior mechanical properties [15, 16]. However, KF has poor water resistance due to the acetyl group in the KGM molecule. The deacetylated KGM reduce the water absorption properties with structure rearrangement from random coil chain to elastic microsphere. This caused KGM to be highly thermostable and forms thermoirreversible gel [17, 18]. Therefore, this research is aimed at studying the properties of KF, which is obtained by dry process extraction of *Amorphophallus muelleri*. It is also aimed at examining the properties of KF specimens subjected to deacetylation and reinforced with microcrystalline cellulose. In addition, it is aimed at investigating the oxalate reduction during the forming process to obtain the properties of KF that would be suitable and applied for making biodegradable food packaging and would increase its commercial value.

## 2. Material and Methods

### 2.1. Materials

*Amorphophallus muelleri* flour was kindly provided by the Union Thai Konjac Co., Ltd. (Tak, Thailand). Microcrystalline cellulose (AR grade) was purchased from SRL Co., Ltd. (Maharashtra, India). Sodium bicarbonate and titanium dioxide (food grade) were supplied by CTi & Science Co., Ltd. (Bangkok, Thailand). Commercial biodegradable packaging was obtained from Environment Co., Ltd. (Bangkok, Thailand).

### 2.2. Methods

#### 2.2.1. Chemical Composition

The general chemical compositions of KF including moisture, ash, insoluble dietary fiber, fat, protein, and carbohydrate were determined using the Association of Official Analytical Chemists International (AOAC) method [19]. The KGM as soluble dietary fiber was determined according to the dinitrosalicylic acid (DNS) method [20]. The starch was calculated by the difference between carbohydrates and total dietary fiber. The amylose was examined by the iodine spectrophotometric method described by Juliano [21].

#### 2.2.2. Physicochemical Properties of KF

*(1) Pasting Properties*. KF (2 g) was mixed with 25 mL of distilled water and then examined by Rapid Visco Analyzer (RVA, NEWPORT Scientific Model VA-4, Australia), as described by Reddy et al. [22]. Pasting parameters (peak time, pasting temperature, peak viscosity, breakdown viscosity, final viscosity, and setback viscosity) were evaluated from the Rapid Visco amylograph.

*(2) Crystallinity*. The crystallinity of KF was investigated by X-ray diffraction pattern according to the modified procedure of Li et al. [23]. KF (1 g) was examined using a Bruker AXS Model D8 Discover X-ray diffractometer (XRD) at 40 kV voltage and 40 mA current and analyzed at 2*θ* ranging from 5° to 50°. The crystallinity index was calculated by the following equation:

(1) $$\begin{array}{c} \text{Degree of crystallinity }\left(\%\right)=\frac{\text{Crystalline area}/\text{Total area}}{\text{Total area}}\times 100. \end{array}$$

*(3) Thermal Properties*. A KF sample (8 mg) was examined by a differential scanning calorimeter (DSC, NETZSCH Model DSC 204 F1 Phoenix, Germany) under a nitrogen atmosphere from 0°C to 450°C at a heating rate of 10°C/min. The thermal properties of KF were assessed using a thermogram according to the modified method of Li et al. [23]

#### 2.2.3. Preparation of KF Specimens Reinforced with MCC

The KF sample of about 5 g was blended with 0, 5, 10, 15, and 20% (w/w) of microcrystalline cellulose (KF/MCC0, KF/MCC5, KF/MCC10, KF/MCC15, and KF/MCC20, respectively). Then, 20% (w/w) of titanium dioxide and 15 mL of 1% sodium bicarbonate solution were added and mixed until homogeneity. The samples were formed at 0.5 mm thickness before heated at 100°C for 10 min and then cooled to room temperature. The samples were flattened by compression moulding at 120°C for 15 min and kept in a desiccator before further analysis.

#### 2.2.4. Properties of KF Specimens Compared with Commercial Biodegradable (CB) Specimen

*(1) Density*. The samples were cut into 25 mm × 50 mm × 0.5 mm before weighing. The experiment was repeated with five different samples [24]. The density was calculated by the following equation:

(2) $$\begin{array}{c} \text{Density }\left(\text{g}/{\text{cm}}^{3}\right)=\frac{\text{specimen mass }\left(\text{g}\right)}{\text{specimen area }\left({\text{cm}}^{2}\right)}\times \text{specimen thickness }\left(\text{cm}\right). \end{array}$$

*(2) Water Absorption Capacity*. The samples were prepared using the same procedure as for density determination. They were weighed (*W*_1_) before water absorption analysis according to the modified method of Debiagi et al. [25] The samples were soaked in 100 mL of distilled water for 1, 5, 10, 15, 20, 25, and 30 min at room temperature, 60°C and 100°C. After that, excess water was removed using tissue paper, and the sample was weighed (*W*_2_). The water absorption capacity was calculated using the following equation:

(3) $$\begin{array}{c} \text{Water absorption capacity }\left(\frac{\text{g water}}{\text{g sample}}\right)=\frac{{\text{W}}_{2}}{{\text{W}}_{1}}. \end{array}$$

The water solution obtained from this analysis was collected to determine soluble oxalate content (see Section 2.2.5.2)

*(3) Mechanical Properties*. The flexural modulus and flexural strength of samples (50 mm × 100 mm × 0.5 mm) were determined using a universal testing machine (HOUNSFIELD Model H10 KM) following the ASTM D790-10 standard at room temperature. Three-point bend testing was conducted at a crosshead speed of 1 mm/min with a load cell of 1 kN. The support span length was 48 mm, and testing was repeated with five replicates [26].

*(4) Thermal Properties*. Thermal properties of the sample were determined by a thermogravimetric analyzer (TGA, Model TG 209 F3 Tarsus, Germany). A 5 mg of sample was heated from 25°C to 600°C with 10°C/min heating rate [9]. From the TGA curve, onset temperature *T*_onset_ (temperature at which the sample starts losing weight) and, from the DTG curve, *T*_max_ (temperature at which the sample lost its maximum weight) were recorded.

Percent change in temperature at which maximum weight loss occurs in the sample was calculated using the following equation:

(4) $$\begin{array}{c} \%\text{Change in }T_{\max}=\left[\frac{\left(T_{\max,\text{treated}}–T_{\max,\text{control}}\right)}{T_{\max,\text{control}}}\right]\times 100, \end{array}$$

where *T*_max,control_ and *T*_max,treated_ are the temperature at which maximum weight loss occurs in the control and treated samples, respectively.

#### 2.2.5. Determination of Soluble Oxalate Content

The soluble oxalate content was determined according to the modified method of Fan [27] and Xu and Zhang [28]. An aliquot of 25 *μ*L of the sample was pipetted into the test tube and then added with 13.7 *μ*L of bromophenol blue, 25 *μ*L of sulfuric acid, 22 *μ*L of potassium dichromate, and 600 *μ*L of deionized water. The mixture was incubated in a water bath at 60°C for 10 min and then added with 55 *μ*L of sodium hydroxide to stop the reaction. The absorbance of the solution was read using a spectrophotometer (Model Lambda EZ201, USA) at 600 nm. Results were calculated as the soluble oxalate using the following equation:

(5) $$\begin{array}{c} \text{Soluble oxalate }\left(\text{mg}/100\text{ g sample}\right)=\frac{\text{soluble oxalate }\left(\text{mg}/\text{mL}\right)\;}{\text{sample concentration }\left(\text{g}/\text{mL}\right)}\times 100. \end{array}$$

*(1) Effects of Forming Process on the Reduction of Soluble Oxalate*. Soluble oxalate content was determined in all samples including KF powder, KF/MCC0, KF/MCC5, KF/MCC10, KF/MCC15, and KF/MCC20. The samples of KF powder (0.05 g) and KF with MCC (2 g) were added with 80 mL deionized water. The solution was heated and kept at 80°C for 15 minutes with continuous stirring. It was then cooled to room temperature and adjusted to volume (100 mL) with deionized water. The solution was centrifuged at 3,000 × g at 25°C for 20 min, and then the supernatant was collected for evaluation of soluble oxalate in KF powder and KF with MCC using the method described in Section 2.2.5.

*(2) Effects of MCC on Preventing the Solubility of Soluble Oxalate*. The water samples obtained from the determination of water absorption capacity of KF with MCC in Section 2.2.4.2 were analyzed for soluble oxalate content following the method described in Section 2.2.5.

#### 2.2.6. Statistical Analysis

Statistical data were analyzed using one-way analysis of variance (ANOVA), and the means were compared by Duncan's multiple range (DMR) using the SPSS (version 21) software at the 95% significance level (*α* = 0.05).

## 3. Results and Discussion

### 3.1. Chemical Composition of KF

KF, a waste material of the KGM industry, contained 65.09% of glucomannan, as the main component. The glucomannan has an acetyl group in the backbone structure that influences the physicochemical properties such as viscosity, gel forming, chemical structure, thermal stability, and especially water absorption capacity [4]. The other components found in KF, included moisture (10.28%), protein (9.43%), ash (7.63%), insoluble fiber (5.99%), and fat (0.45%) as shown in Table 1. In addition, starch content found was 1.06% with high in amylopectin. The amount of protein found in KF was higher than the specification of the European Food Safety Authority (EFSA), which defines less than 8% protein [6]. Since the protein composition in this study was calculated based on total nitrogen content, the protein content of KF may combine the amino acids and nitrogen compounds such as trimethylamine. The trimethylamine causes a fish-like smell, which is a unique odor in KF [29].

### 3.2. Physiochemical Properties of KF

#### 3.2.1. Pasting Properties

**Figure 1:**
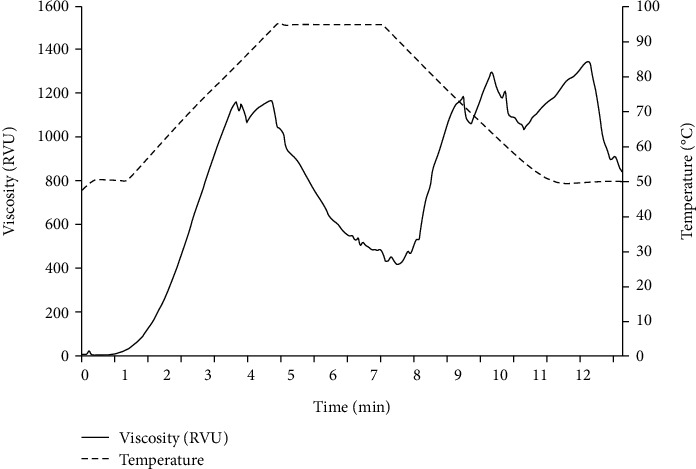
Pasting profile of konjac flour (8% w/w concentration).

Results in Figure 1 show that KF had a pasting temperature at 55.75°C. During gelatinization, KF had peak viscosity at 4 min and 1254.97 RVU while hold viscosity and breakdown viscosity of KF were 396.55 and 858.43 RVU, respectively. The high peak and breakdown viscosity indicated that the granule structure had a low thermal stability and shear resistance. Final viscosity and setback viscosity were 839.30 and 442.76 RVU, respectively. The high final and setback viscosity indicated that starch paste was rigid and stable.

Comparing to common biopolymers such as cassava and corn starch, which were described by Srichuwong et al. [30], it appears that KF had a lower pasting temperature and peak time than cassava and corn starch. In addition, KF had a higher peak viscosity, breakdown viscosity, final viscosity, and setback viscosity than cassava and corn starch. Since the acetyl group leads to high water absorption and swelling [31], during heating, viscosity of KF increased to maximum viscosity and the structure of granules broke down, resulting in a higher density KGM molecules. The result might be attributed to KGM molecules being arranged and forming a semisolid network structure, resulting in a layer with higher viscosity on the surface of the molecule. Upon cooling, KGM aggregated in a continuous phase and formed a rigid and stable gel [32]. This result is consistent with the findings of Ma et al. [33] that glucomannan enhances starch retrogradation to form a strong gel. Hence, the water molecules could easily interact with glucomannan KF. This phenomenon is related to its pasting properties to show that KF can rapidly swell and attain high viscosity.

#### 3.2.2. Crystallinity

**Figure 2:**
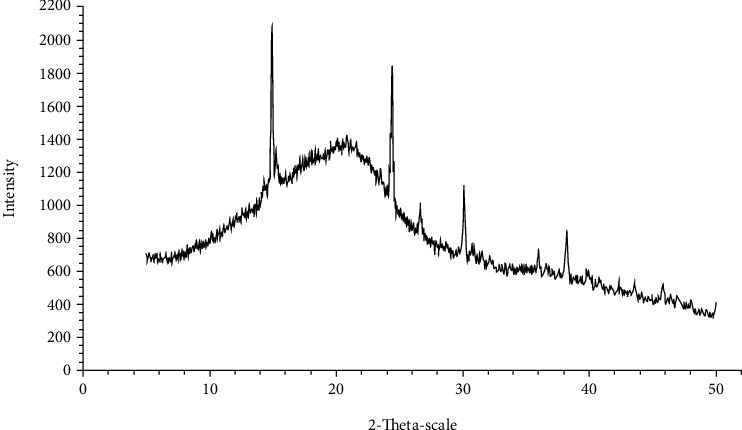
X-ray diffraction pattern of konjac flour.

The crystallinity of KF is presented in Figure 2. Result shows that KF had 6.6% crystallinity. This indicates that KF structure comprises some crystalline regions due to the main component of KGM, which has mostly amorphous structure [34, 35]. In addition, the structure of KF was loosely packed and disorganized as indicated by a few peaks shown in Figure 2. Comparing to cassava and corn starch, it appears that KF in this work had a lower amylose content of 0.24% (Table 1), while cassava and corn starch had 17.9% amylose and 23.4% amylose, respectively [36]. The amylose content is related to A-type crystallinity of KF, which arranges tightly packed structures [36].

#### 3.2.3. Thermal Properties

**Figure 3:**
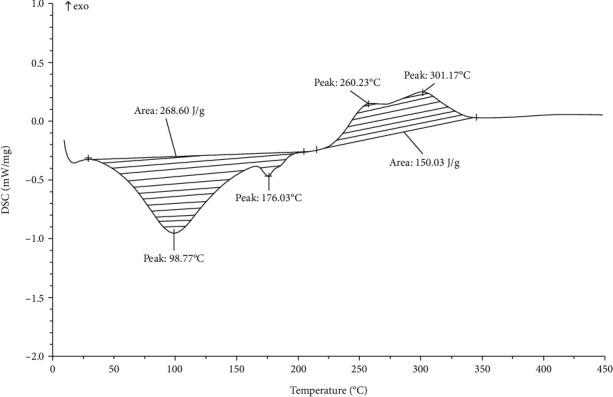
Thermogram DSC of konjac flour.

When KF is tested using a differential scanning calorimeter (DSC), several thermal properties can be observed. The DSC is an analytical instrument that measures the heat flow into or out of a sample as a function of temperature or time. DSC measures temperatures of a reference material and a sample while changing the sample temperature in accordance with a program, and then measures the amount of heat from the temperature difference. The thermogram of KF shown in Figure 3 reveals that KF had two phase transitions. The first transition was endothermic (melting transition). The second transition was exothermic (decomposition transition) of KGM polymer chains [37, 38]. The DSC can determine the temperature at which KF undergoes a phase transition from solid to liquid, known as the melting temperature. This transition is associated with the breaking of intermolecular forces and changes in the crystalline structure of KF. In Figure 3, it was found that KF thermogram had double peaks in both melting and decomposition transition. The maximum melting and decomposition temperature at the first peak was the KGM transition, and the second peak was the calcium oxalate transition. In the initial stage, an endothermic reaction or melting occurs, leading to the dissociation of bonds between glucomannan molecules and water. This is due to the presence of a significant amount of hydroxyl groups, which are abundant in glucomannan present in konjac flour. These hydroxyl groups exhibit strong attractive forces with water molecules, resulting in high moisture content in the flour. Consequently, it is possible that during the early stage of the reaction (the first peak), there is a loss of moisture (dehydration) within the molecular structure of the konjac flour. These results are in accordance with reports of Wang et al. [35] and Lozano et al. [39] The results indicated that KF had a melting temperature of 98.77°C and a decomposition temperature of 260.23°C, while calcium oxalate melted at 176.03°C and decomposed at 301.17°C. The melting and decomposition energies of both substances were 268.60 and 150.03 J/g, respectively.

The melting temperature of calcium oxalate can vary depending on the specific crystal structure and purity of the compound. However, in general, calcium oxalate does not have a well-defined melting point because it decomposes before it reaches a true liquid state. Calcium oxalate undergoes thermal decomposition into calcium carbonate (CaCO_3_) and carbon monoxide (CO) when heated. This process typically occurs around temperatures of 200°C for the anhydrous form of calcium oxalate [40]. The decomposition temperature may be lower if the compound contains water of hydration. It is worth noting that calcium oxalate exists in different hydrate forms, such as monohydrate (CaC_2_O_4_·H_2_O) and dihydrate (CaC_2_O_4_·2H_2_O). The presence of water molecules can affect the thermal behavior and decomposition temperature of these hydrates.

Comparing to the melting transition of cassava and corn starch described by Srichuwong et al. [36], it was found that KF had a lower onset temperature (31.12°C), in which cassava flour showed 59.3°C, while corn starch showed 62.6°C of onset temperature. This was due to the lower crystallinity of KF leading to rapid dehydration in the initial melting period [41]. Meanwhile, KF had higher peak temperatures and enthalpy than cassava and corn starch when the temperature increased. As a result of the hydrophilicity of KGM molecule, the KF swelled and slowly became dehydrated [38]. Therefore, the KF had higher thermal stability for melting than cassava and corn starch. These properties in KF support its potential for the development of biodegradable packaging. The suitable characteristics are the ability of KF to form a resistant gel and its thermal stability, which are important for the forming process. However, KF has a low crystallinity, which favours hydration. This can limit its applications, so these properties must be improved by deacetylation and reinforcement with MCC to obtain suitable properties for food packaging.

### 3.3. Characteristics of KF Specimen Reinforced with MCC

#### 3.3.1. Physical Properties of KF Specimens

*(1) Thickness and Density*. The thickness of KF, KF/MCC at all concentrations, and CB was in a range of 0.51-0.53 mm, and it had no significant differences (*p* > 0.05). In addition, the density of all specimens was 0.88-0.91 g/cm^3^. It showed no significant difference (*p* > 0.05) between KF and KF/MCC at all concentrations. However, the samples had a significantly higher density than CB which was 0.68 g/cm^3^ (*p* ≤ 0.05). KF and KF/MCC at all concentrations had lower volume than CB because they were modified by deacetylation to eliminate disorderly group in the glucomannan backbone, resulting in a tightly organized structure [17, 42]. In contrast, CB was composed of the bagasse pulp mixed with a bamboo pulp containing cellulose, hemicellulose, and lignin, resulting in disorganized CB structure with gaps in the molecular structure [43, 44].

**Figure 4:**
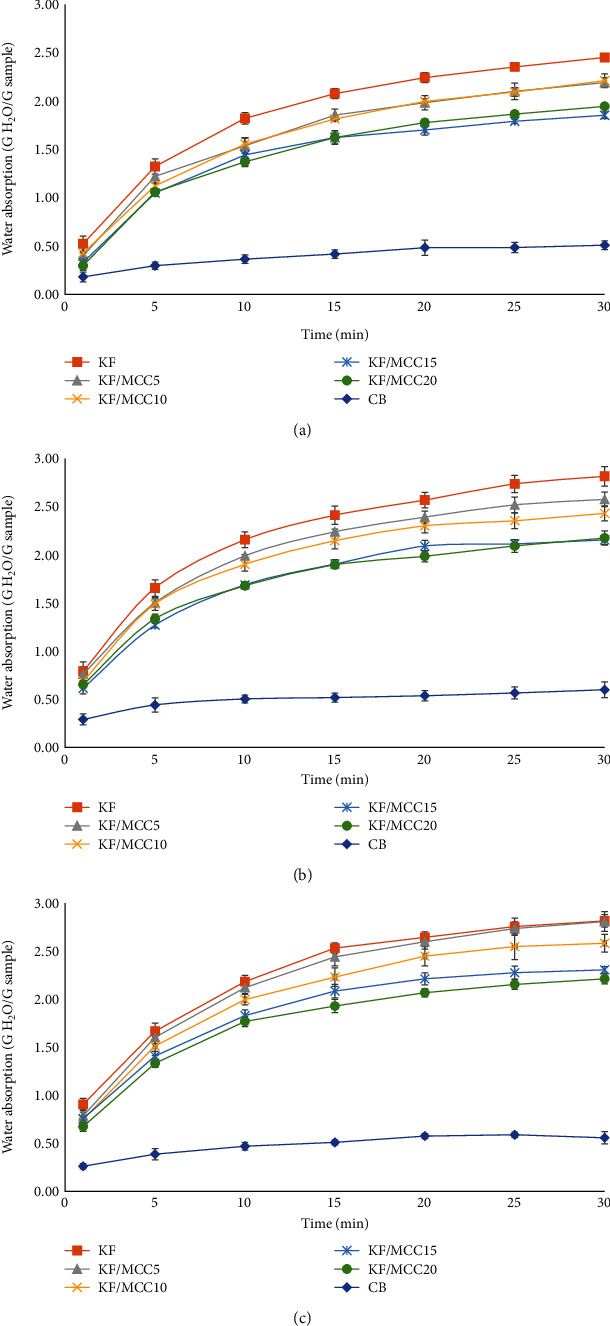
Water absorption of specimens: (a) room temperature, (b) 60°C, and (c) 100°C.

*(2) Water Absorption*. Water absorption of specimens at room temperature (27°C) is presented in Figure 4(a). KF/MCC15 and KF/MCC20 had a lower water absorption than KF/MCC10, KF/MCC5, and KF. This was due to the highly crystalline structure of MCC, which is closely packed within hydrogen bond network [45]. Thus, MCC replaced the amorphous parts of KF and enhanced water resistance. Moreover, KF and MCC have similar chemical structures, which include glucose [46], resulting in an excellent bonding between KF and MCC. This is possibly because MCC was hygroscopic and free hydroxyl groups on the surface of MCC molecules interacted with the water [47]. The KF/MCC specimens showed higher flexibility with increasing water absorption time. The MCC molecules became rearranged and agglomerated resulting in decreased dispersion of MCC in KF and reduced reinforcement efficiency [11, 48]. This result was related to the water absorption at 60°C (Figure 4(b)). However, the treatment at 100°C (Figure 4(c)) showed that KF reinforced with 20% MCC exhibited higher water resistance than KF/MCC15, KF/MCC10, KF/MCC5, and KF specimens. This was a result of high temperature inducing the stronger interaction of the free hydroxyl group of MCC with the side chain of KGM providing stronger binding between KGM and MCC. However, the water resistance of KF reinforced with 15% and 20% MCC was lower than that of the CB specimen due to the positive effects of the acetyl group of KGM and the free hydroxyl group of MCC on water absorption. Moreover, the CB specimen contained a water-insoluble polymer [49].

#### 3.3.2. Mechanical Properties

The mechanical properties of specimens are shown in Table 2. Results reveal that the flexural strength of KF and all KF/MCC specimens showed no significant differences (*p* > 0.05). It was found that KF/MCC5 sample had higher flexural strength than the KF sample due to the crystalline particles of MCC. However, strengthening with MCC at high concentrations decreased the flexural strength as observed in KF/MCC20. This result is consistent with previous studies, which found that the mechanical properties of specimens decreased with increasing MCC contents due to agglomeration [48, 50]. Yet, the KF specimen had higher flexural strength than the CB specimen. This may be attributed to the structure of the KF specimen that was more compact than the CB specimen, which was reflected by the higher density as reported earlier.

However, the flexural modulus of KF, KF/MCC, and CB specimens showed no significant difference (*p* > 0.05). When microcrystalline cellulose (MCC) is added to a material, it can have varying effects on the flexural modulus depending on several factors, including the amount of MCC added, the dispersion and distribution of MCC within the material, and the specific properties of the base material. In some cases, the addition of MCC may not significantly alter the flexural modulus of the material. This can occur when MCC is added in relatively small amounts or when the MCC particles are not uniformly dispersed within the material. In such cases, the MCC particles may not contribute significantly to the overall stiffness of the material, and the flexural modulus remains relatively unchanged. Additionally, if the base material already possesses a high flexural modulus, the addition of MCC might not have a noticeable impact on the overall stiffness of the material. In such cases, the inherent stiffness of the base material can dominate the mechanical properties, and the contribution of MCC to the flexural modulus might be minimal. However, it is important to note that the impact of MCC on the flexural modulus can vary depending on the specific material system and processing conditions. In other instances, the addition of MCC can indeed affect the flexural modulus, especially if larger amounts of MCC are incorporated or if the MCC particles are well-dispersed and interact with the matrix material, reinforcing it and increasing its stiffness.

#### 3.3.3. Thermal Properties

Thermal properties of KF, KF/MCC, and CB specimens are characterized by two degradation stages. The first degradation stage is the loss of moisture and other components with low molecular weights. The second degradation stage is the loss of the major components. The maximum degradation temperature and weight loss of KF, KF/MCC at various concentrations, and CB specimens are shown in Table 3. The initial thermal decomposition showed that KF and KF/MCC at all concentrations had weight loss between 16.9 and 18.7%, while the maximum degradation occurred at temperatures around 250°C, which agreed with the findings by Nguyen Tien et al. [51]. KF and KF/MCC at all concentrations had higher thermal degradation and more weight loss than the CB specimen. Since KGM has good water absorption and holding capacity [4, 52], water from KF specimens was evaporated at a higher temperature than that from CB specimens.

As for the second degradation, the KF specimen had a maximum degradation temperature at 295.9°C, which was close to the maximum degradation of KGM. The weight loss of KGM at this heating stage could be attributed to the degradation of polysaccharide rings and disintegration of macromolecule chains, resulting in the separation of main chains of KGM molecules [51]. The reinforcement with MCC to KF specimens promoted the thermal stability of the KF specimens. KF/MCC20 had a higher degradation temperature than KF/MCC15, KF/MCC10, and KF/MCC5. This degradation was caused by the dissociation of KGM and MCC. The KGM molecule was decomposed at a lower temperature than MCC. This was due to the amorphous form mainly present and a high number of oxygen groups in KGM molecules [53]. Moreover, the MCC has a maximum degradation at 300°C [54], which is similar to KF/MCC20. Therefore, increasing the MCC content enhanced the thermal stability of KF specimen. However, KF/MCC20 had a lower thermal stability than CB specimen due to a large amount of oxygen groups in KGM.

### 3.4. Soluble Oxalate Content

#### 3.4.1. Effects of Packaging Forming Process on the Reduction of Soluble Oxalate

The soluble oxalate contents in KF powder and all KF specimens are shown in Table 4. The soluble oxalate content in KF powder was 131.95 mg/100 g sample, whereas KF and KF/MCC specimens of all concentrations had less than 70 mg/100 g packaging material sample made from native KF powder. This indicates that the forming process consisting of wet heat treatment followed by heating compression could reduce the soluble oxalate. This result agrees with the reports of Chai and Liebman [55] and Bong et al. [56] revealing that soluble oxalate was easily eliminated during heating, especially boiling and steaming. Because the soluble oxalate could be dissolved in water and eliminated with the water vapor, no significant differences of oxalate reduction in all KF/MCC specimens were found (Table 4).

#### 3.4.2. Effects of MCC Reinforcement on the Solubility of Soluble Oxalate

**Figure 5:**
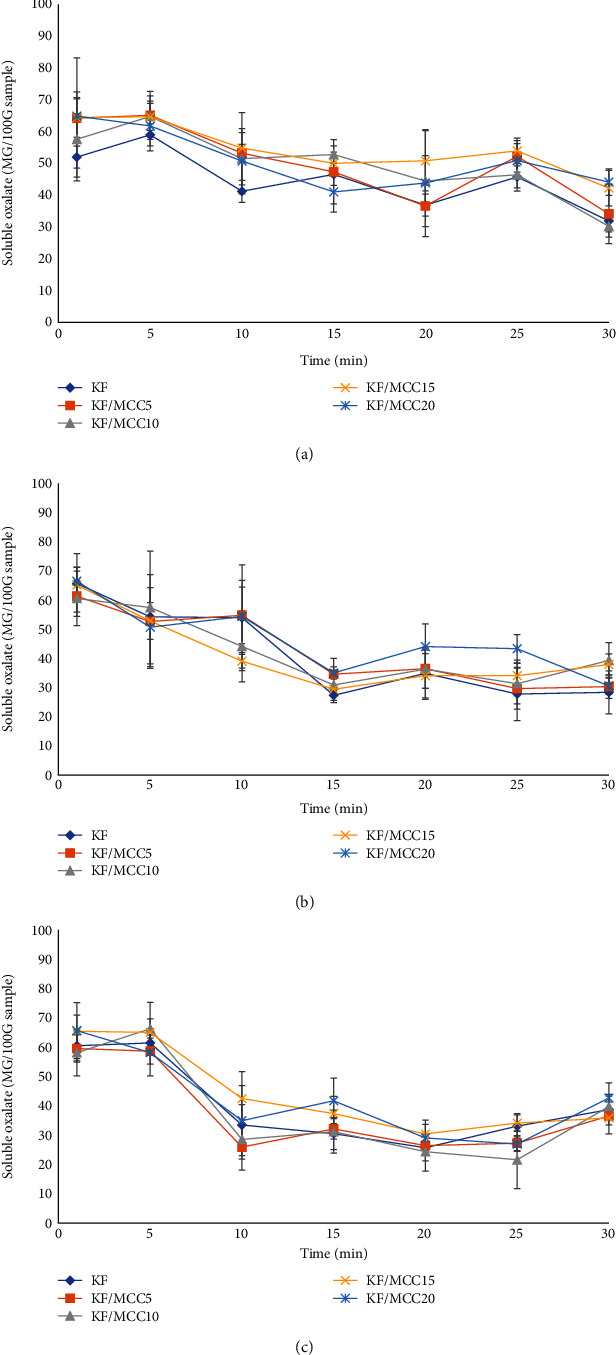
Solubility of soluble oxalate in specimens: (a) room temperature, (b) 60°C, and (c) 100°C.

The soluble oxalate contents in water samples obtained from water absorption analysis at room temperature, 60°C and 100°C, are presented in Figure 5. It was observed that all samples exhibited a solubility of soluble oxalate not exceeding 65 mg/100 g. Upon analyzing the effects of varying concentrations of MCC, it was found that the solubility of soluble oxalate did not differ significantly, indicating that the addition of MCC has no impact on preventing the dissolution of soluble oxalate from the sample. Additionally, when considering the immersion time of the test samples in water, it was observed that the highest solubility of soluble oxalate occurred within the first 5 minutes of testing, followed by a decreasing trend as the immersion time of the test samples increased. However, this change was not statistically significant (*p* > 0.05), suggesting that during the initial stages of the test, there might not have been sufficient water absorption by the test samples, leading to the release of soluble oxalate from the sample surface. However, as the immersion time increased and the test samples' starch component, which contains easily absorbable glucomannan [57–59], swelled, the test samples were able to reabsorb the soluble oxalate from the test water, resulting in a decrease in the detected amount of soluble oxalate in the test water. Nonetheless, based on the water absorption test results conducted on the samples, it can be inferred that if the test duration exceeds 30 minutes, the solubility of soluble oxalate in the water tends to decrease over time. In this experiment, a test duration of 30 minutes was chosen to simulate the time frame of consumer packaging usage and the appropriate duration for consuming a meal, typically ranging from 20 to 30 minutes.

Oxalate is a fine particulate impurity commonly found in konjac flour. There are two forms of oxalate in KF: water-soluble oxalate and water-insoluble crystalline oxalate [60]. The effect of temperature on the solubility of oxalates can vary depending on whether they are soluble or insoluble in water. For soluble oxalates, an increase in temperature generally leads to an increase in solubility. This is because higher temperatures provide more energy to break the intermolecular forces holding the oxalate ions together, allowing them to dissolve more readily in the solvent. On the other hand, insoluble oxalates tend to have very low solubility in water regardless of temperature. Their solubility does not significantly change with temperature variations within a typical range. In this case, based on experimental results, it was found that the amount of dissolved oxalate in water at a temperature of 100°C is lower than that in water at room temperature. It can be inferred that increasing the temperature of the water tends to decrease the solubility of the oxalate, resulting in less dissolution.

## 4. Conclusion

Low-grade common KF is a waste material generated during purified KGM production. It is obtained by dry extraction of tubers of several species of *Amorphophallus*, particularly *A. muelleri*. The waste contains KGM molecules with various sizes, impurities, and oxalate salts. The main component is KGM, which to a large extent influences the physicochemical properties of KF. Although KF has lower crystallinity, it has higher thermal stability and pasting properties than cassava and corn starch. Modification of KF structure by deacetylation and reinforcement with MCC can improve the water resistance of KF. Present study showed that MCC enhanced water and thermal resistance; however, it was not as good as CB obtained from the bagasse pulp mixed with bamboo pulp. The forming process reduced soluble oxalate in the specimens by 53.50% as compared to native KF powder. All KF specimens had a solubility of soluble oxalate not exceeding 65 mg/100 g sample.

KF has the potential to be used as a biodegradable material for food packaging. However, it still requires further studies on structure modification or the addition of another reinforcing agent to improve the water resistance. Furthermore, additional studies are required to reduce oxalate content in KF. The use of KF would not be only to manufacture food packaging and minimize the use of plastics but also add value to the processing waste produced during the KGM purification.

## Figures and Tables

**Table 1:** Chemical composition of konjac flour.

Composition	Content (% ± s.d.)
Total carbohydrate	72.14 ± 0.04
(i) Soluble fiber (glucomannan)	65.09 ± 0.06
(ii) Insoluble fiber	5.99 ± 0.16
(iii) Amylopectin	0.82 ± 0.09
(iv) Amylose	0.24 ± 0.03
Moisture	10.28 ± 0.02
Protein	9.43 ± 0.03
Ash	7.63 ± 0.06
Fat	0.45 ± 0.02

**Table 2:** Flexural strength and flexural modulus of specimens.

Sample	Flexural strength (N/mm^2^)	Flexural modulus (N/mm^2^)^ns^
KF	50.26 ± 9.33^a^	4,524.80 ± 1,099.89
KF/MCC5	56.68 ± 9.77^a^	5,086.80 ± 1,141.83
KF/MCC10	50.88 ± 7.39^a^	4,605.20 ± 765.88
KF/MCC15	52.74 ± 1.78^a^	4,928.20 ± 452.24
KF/MCC20	47.04 ± 5.93^a^	4,221.20 ± 832.63
CB	33.13 ± 4.86^b^	4,336.64 ± 755.57

Means within a column followed by different superscript letters (a, b) are significantly different (*p* ≤ 0.05); ns: not significant.

**Table 3:** Thermal properties of konjac flour specimen compared with the commercial biodegradable specimen.

Sample	First stage		Second stage	
	*T*_max_ (°C)	Weight loss (%)	*T*_max_ (°C)	Weight loss (%)
KF	250.3	18.68	295.9	39.45
KF/MCC5	249.7	17.78	295.0	41.18
KF/MCC10	251.3	18.04	297.1	41.38
KF/MCC15	251.5	16.91	297.3	42.87
KF/MCC20	251.6	17.21	300.6	43.04
CB	69.5	6.05	357.4	80.59

*T*_max_: maximum degradation temperature.

**Table 4:** Soluble oxalate content of konjac flour and konjac flour specimens.

Samples	Soluble oxalate (mg/100 g DW)
KF powder	131.95 ± 3.56^a^
KF specimen	61.36 ± 2.82^b^
KF/MCC5	62.75 ± 1.16^b^
KF/MCC10	65.56 ± 1.59^b^
KF/MCC15	61.78 ± 0.27^b^
KF/MCC20	63.23 ± 2.16^b^

Means within a column followed by different superscript letters (a, b) are significantly different (*p* ≤ 0.05).

## Data Availability

The research data used to support the findings of this study are available from the corresponding author upon request.
